# Using RSI and RFS scores to differentiate between reflux-related and other causes of chronic laryngitis

**DOI:** 10.1016/j.bjorl.2021.08.003

**Published:** 2021-10-17

**Authors:** Claudia Alessandra Eckley, Rodrigo Tangerina

**Affiliations:** Fleury Medicina e Saúde Laboratórios de Diagnóstico, Divisão de Otorrinolaringologia, São Paulo, SP, Brazil

**Keywords:** Laryngopharyngeal reflux, Gastroesophageal reflux disease, Obstructive sleep apnea, Allergic rhinitis, Diagnosis

## Abstract

**Objective:**

To establish if the Reflux Symptom Index (RFI) and the Reflux Finding Score (RFC) can help establish the differential diagnosis in patients with distinct causes of chronic laryngopharyngitis.

**Methods:**

A group of 102 adult patients with chronic laryngopharyngitis (Group A – 37 patients with allergic rhinitis; Group B – 22 patients with Obstructive Sleep Apnea (OSA); Group C – 43 patients with Laryngopharyngeal Reflux (LPR)) were prospectively studied. Chronic laryngitis was diagnosed based on suggestive symptoms and videolaryngoscopic signs (RSI ≥ 13 and RFS ≥ 7). Allergies were confirmed by a positive serum RAST, OSA was diagnosed with a positive polysomnography, and LPR with a positive impedance-PH study. Discriminant function analysis was used to determine if the combination of RSI and RFS scores could differentiate between groups.

**Results:**

Patients with respiratory allergies and those with LPR showed similar and significantly higher RSI scores when compared to that of patients with OSA (*p* < 0.001); Patients with OSA and those with LPR showed similar and significantly higher RFS scores when compared to that of patients with Respiratory Allergies (OSA vs. Allergies *p* < 0.001; LPR vs. Allergies *p* < 0.002). The combination of both scores held a higher probability of diagnosing OSA (72.73%) and Allergies (64.86%) than diagnosing LPR (51.16%).

**Conclusions:**

RSI and RFS are not specific for reflux laryngitis and are more likely to induce a false diagnosis if not used with diligence.

## Highlights

• Reflux laryngitis is highly prevalent in the adult population sharing symptoms of laryngeal and pharyngeal inflammation with a number of inflammatory, infectious and traumatic conditions.
• Diagnosis of Laryngopharyngeal Reflux is challenging and mainly based on suggestive symptoms and videolaryngoscopic signs of inflammation. Objective tests, such as prolonged esophageal ph-metry and impedance-pHmetry, are costly and have low sensitivity.
• To minimize the subjectivity of clinical diagnosis for Laryngopharyngeal Reflux a number of scores have been proposed, being the most widely used the Reflux Symptom Index (RSI) and the Reflux Finding Score (RFS).
• The current study used the RSI and the RFS to try to differentiate between the most common causes of inflammatory and traumatic chronic pharyngitis and laryngitis.

## Introduction

Chronic laryngopharyngitis is commonly associated to upper respiratory complaints, such as sore throat, cough, throat clearing, difficulties swallowing and hoarseness.^1^, ^2^, ^3^ It may be caused by a number of allergic, inflammatory or infectious agents.^3^ The differential diagnosis is a constant challenge, especially because many of these etiologies may co-exist.

Lifestyle in the post-modern world has incurred in social habits that are notoriously unhealthy. Living in large, polluted cities has increased the number of individuals suffering of respiratory allergies.^4^, ^5^, ^6^, ^7^ Unbalanced meals and poor eating habits lead to weight gain. It is also well known that obese patients are more likely to snore and have Obstructive Sleep Apnea (OSA).^8^, ^9^, ^10^, ^11^ The collapse of the upper airway leading to friction of the mucosa of the pharynx, palate, tongue and larnynx, may also cause mucosal injury mimicking an inflammatory/infectious event.^8^, ^10^ Obesity changes the immune response, and also facilitates diseases such as Gastroesophageal Reflux Disease (GERD) and its supraesophageal manifestations (Laryngopharyngeal Reflux – LPR).^7^, ^8^, ^9^, ^10^, ^11^

A very special focus in studying the role of the proximal ascent of gastroduodenal contents into the upper airways has developed in past decades, as can be observed by the enormous amount of literature on the topic.^1^, ^2^, ^3^, ^12^, ^13^, ^14^ Most specialists find LPR to be a major player in chronic laryngeal and pharyngeal inflammation.^2^ However, the excessive importance given to reflux has partially confused the medical community, whereas it is now believed that the disease has been over diagnosed and over treated incurring in great costs in drugs and its significant side effects.^1^, ^3^, ^12^

Part of the difficulties arise from the lack of a cost-effective, non-invasive objective methods to diagnose LPR.^1^, ^2^ Prolonged esophageal monitoring, although specific, has a poor sensibility.^1^, ^12^, ^13^ Saliva pepsin tests seem promising, but have not been fully validated by the scientific community.^13^, ^14^ Most clinicians rely on suggestive symptoms and inflammatory signs observed during flexible or rigid laryngoscopies.^1^, ^2^, ^3^, ^12^, ^13^

Instruments have been developed to decrease the subjectivity of clinical diagnosis. The most widely used throughout the world are those proposed by Belafsky et al., the Reflux Symptom Index^15^ and the Reflux Finding Score.^16^ Positive scores, especially when combined, have warranted clinical treatment of LPR in the lack of objective diagnostic tests.^1^, ^2^, ^3^, ^12^, ^17^ However, over the years it has been observed that these instruments are widely non-specific and should be used with caution.^1^, ^12^, ^17^

The objective of the current study was to try to establish if the Reflux Symptom Index and the Reflux finding score can help establish the differential diagnosis in patients with distinct causes of chronic laryngopharyngitis.

## Methods

A group of 102 adult patients with chronic laryngopharyngitis were consecutively enrolled over a six-month period after properly consented. The study was conducted in accordance with the principles of the Declaration of Helsinki. Approval was granted by the Institutional Ethics Committee for Research in Humans (IFSP#3.244.948).

The study group was subdivided in three distinct sub-groups that were carefully selected to avoid overlap in diagnosis: Group A – 37 patients with allergic rhinitis; Group B – 22 patients with OSA; Group C – 43 patients with LPR.

In order to avoid the bias of other causes of chronic laryngitis, smokers and drinkers, as well as those reporting vocal abuse or misuse, infectious rhinosinusitis, prior surgery/radiation to the head and neck, as well as those using inhaled steroids were excluded.

General inclusion criteria were: age between 18 and 75 years, both genders, BMI < 35, symptoms and videolaryngoscopic signs of chronic laryngitis and pharyngitis.

Chronic laryngopharyngitis was diagnosed in patients with complaints of sore throat, scratchy throat, hoarseness, throat clearing, post nasal drip, thick mucus in the throat without fever, malaise or other systemic symptoms, with the duration of over 12 weeks.^1^, ^2^, ^14^ Upon physical examination the patient should present with at least three of the following inflammatory sings at the pharynx and larynx: hyperemia and/or lymphoid hyperplasia of the posterior pharyngeal wall/and or pharyngeal, palatine and/or lingual tonsils, edema and/or hyperemia of the laryngeal mucosa, especially of the arytenoids and posterior cricoid area.^1^, ^14^

All subjects underwent objective diagnostic tests for Respiratory Allergies (RAST), OSA (polysomnography) and GERD/LPR (Multichannel Intraluminal Impedance-pH esophageal monitoring – MII, and/or Esophagogastroduodenoscopy – EGD).

Respiratory allergies were established during medical history by inquiring patients about previously diagnosed allergic rhinitis or asthma/bronchitis, such as sneezing, clear nasal discharge, nasal itching and obstruction, cough, and wheezing, triggered by inhaled allergens). These symptoms were then corroborated by a positive blood work-up for specific IgE (RAST panels) for inhaled allergens).

OSA was diagnosed based on a history of snoring and witnessed apnea, daytime sleepiness, and a positive Berlin test, corroborated by a polysomnography with apnea/hypoxia index >5.

LPR was diagnosed by a positive objective test such as prologed 24 h esophageal monitoring (double channel pH-metry or pHimpedance monitoring), and/or an,EGD with erosive esophagitis grade B or C, or Barret’s esophagus.

Specific inclusion criteria for each sub-group was:

Group A– A history of respiratory allergies corroborated by a positive RAST test for respiratory allergens, and no complaints or positive tests for OSA or GERD/LPR.

Group B– A history of snoring and obstructive sleep apnea, corroborated by an Epworth slippiness scale greater >10,^18^ a standard Polysomnography (PSG) with Apnea/Hypopnea Index (AHI) >5,^19^ and no complaints or positive tests for respiratory allergies or GERD/LPR.

Group C– A negative history and tests for respiratory allergies and OSA, clinical signs of chronic laryngopharyngitis corroborated by a positive esophageal impedance-pHmetry and/or Esophagogastroduodenoscopy (EGD).

All subjects filled out the Reflux Symptom Index (RSI), a 13 sub-domain questionnaire on symptoms associated to LPR, considered positive if above 13 points.^15^ Subjects were also submitted to flexible nasal endoscopy to establish the Reflux Finding Score (RFS), a 9-sub-domain scoring system based on inflammatory endolaryngeal findings, considered positive if above 7 points.^16^ All exams were recorded and scored by two independent and experienced otolaryngologists, who were blinded to the clinical history. In order to establish test-retest reliability, each video was rated twice with an interval of more than 24 h and less than 72 h by each of the rates.

Respiratory allergies were confirmed through blood samples with a positive RAST for respiratory allergens (Total IGE > 183,0 UI/mL).^20^ Sleep studies were conducted in a routine fashion following previously established protocols, and considered positive for OSA if AHI > 5 (Alice 5 Respironics; Murrysville, PA).^19^ Reflux was confirmed by EGD and Multichannel Intraluminal Impedance-pH esophageal monitoring (MII) (Sandhill Scientific; Highlands Ranch, CO), where distal pathological reflux was diagnosed according to De Meester scores and proximal pathological reflux using the RAI score.^1^, ^3^, ^17^ Esophagogastroduodenoscopy was considered positive for GERD in the presence of erosive esophagitis and Barret’s esophagus.^21^

Data were expressed as mean for variables with normal distribution and median for variables with non-normal distribution. Multiple group comparisons were performed using one-way analysis of variance (ANOVA), followed by Chi-Square test and Fisher’s exact test, when applicable. Intra and interrater reliability was studied using the Kappa score. Discriminant function analysis, a multivariate statistical method that serves to set up a model to predict group memberships, was used to determine if RSI and RFS combined could discriminate between groups of patients with distinct causes of chronic laryngitis. Significance level was established at *p* < 0,05.

## Results

The mean age of subjects with Respiratory Allergies (Group A) was 48.16 ± 13.10 years; 11 were males and 26 were females. In the OSA group (Group B), 17 were males and 5 were females with a mean age of 52.09 ± 11.82 years. Subjects in the GERD/LPR group (Group C) had a mean age of 53.60 ± 12.65; 23 were males and 20 were females (Table 1, Table 2). No statistically significant difference was observed in the mean age of patients between the study groups. As for gender, a significant difference was observed in the males/female ratio between the OSA and Allergy groups, with predominance of males in the former and of females in the latter (Table 1).

**Table 1:** Group of patients with chronic pharyngolaryngitis sub-divided into three etiological groups according to gender.

	Group			Total	*p*
	Allergies	OSA	LPR		
	n (%)	n (%)	n (%)	n (%)	
Female	26 (70.27)	5 (22.73)	20 (46.51)	51 (50.00)	0.001^a^*(*Fisher's exact test*)*
Male	11 (29.73)	17 (77.27)	23 (53.49)	51 (50.00)	
Total	37 (100)	22 (100)	43 (100)	102 (100)	

OSA, Obstructive Sleep Apnea; LPR, Laryngopharyngeal Reflux; significance level *p* ≥ 0.005.

**Table 2:** Group of patients with chronic pharyngolaryngitis sub-divided into three etiological groups according to age.

Group	n	Age (in years*)*			
		Mean	SD	Median	*p*
Allergies	37	48.16	13.10	47.00	0.155 (ANOVA)
		(44.05, 52.21)		(43.00, 48.00)	
OSA	22	52.09	11.82	51.00	
		(47.32, 56.86)		(48.00, 54.50)	
LPR	43	53.60	12.65	57.00	
		(50.07, 57.21)		(53.00, 59.00)	

OSA, Obstructive Sleep Apnea; LPR, laryngopharyngeal Reflux; SD, Standard Deviation; significance level *p* ≥ 0.005.

**Figure 1:**
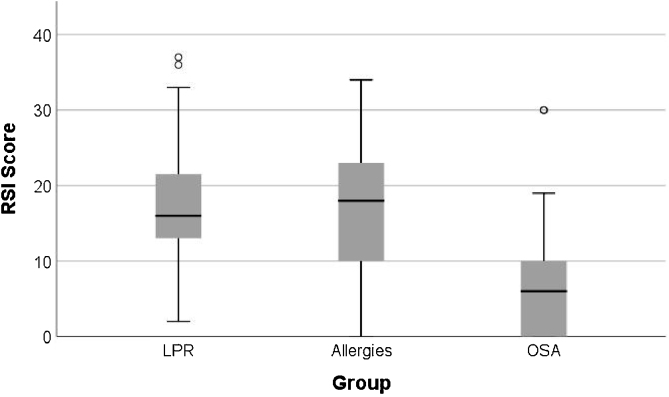
Box plot comparing RSI score between patients in the three sub-groups with chronic pharyngolaryngitis.

Inter and intra-rater variability was insignificant between both examiners for both RSI and RFS scores (Kappa 0.8 for both), thus it was opted to use the average scores for final analysis,

The mean RSI scores in the Allergy group was 16.38 ± 9.18, in the OSA group it was 7.59 ± 8.81, and in the LPR group it was 16.91 ± 8.40 (Table 3). Patients with respiratory allergies and those with LPR showed similar and significantly higher RSI scores when compared to that of patients with OSA (*p* < 0.001) (Fig. 1).

**Table 3:** Reflux Symptom Index (RSI) scores of patients with chronic pharyngolaryngitis sub-divided into three etiological groups.

Variable	Group	n	Mean	SD	Median	Min.	Max.	*p*	post hoc	*p*	T.E.
RSI	LPR	43	16.91	8.40	16.00	2.00	37.00	<0.001^a^	LPR vs. Allergies	>0.999	0.018
			[14.42, 19.65]		[15.00, 17.00]						
	Allergies	37	16.38	9.18	18.00	0.00	34.00		LPR vs. OSA	<0.001^a^	0.483
			[13.41, 19.33]		[13.00, 20.00]						
	OSA	22	7.59	8.81	6.00	0.00	30.00		Allergies vs. OSA	0.001^a^	0.477
			[4.54, 11.10]		[3.00, 7.00]						

OSA, Obstructive Sleep Apnea; LPR, Laryngopharyngeal Reflux; SD, Standard Deviation; min, minimum; max, maximum; significance level *p* ≥ 0.005.

**Figure 2:**
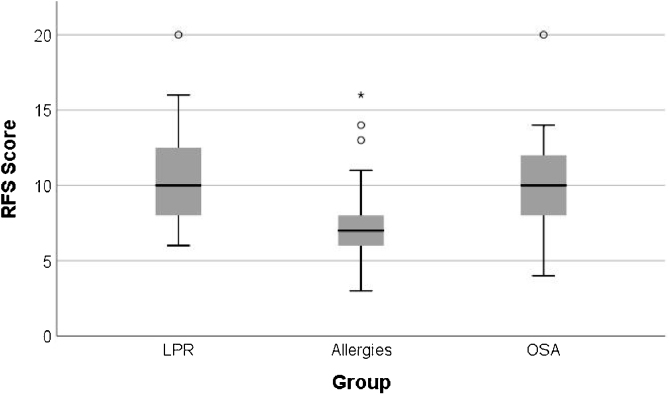
Box plot comparing RFS score between patients in the three sub-groups with chronic pharyngolaryngitis.

The mean RFS scores in the Allergy group was 7.49 ± 2.72, in the OSA group it was 10.23 ± 3.32, and in the LPR group it was 10.65 ± 3.04 (Table 4). Patients with OSA and those with LPR showed similar and significantly higher RFS scores when compared to that of patients with Respiratory Allergies (OSA vs. Allergies *p* < 0.001; LPR vs. Allergies *p* < 0.002) (Fig. 2).

**Table 4:** Reflux Finding Score (RFS) scores of patients with chronic pharyngolaryngitis sub-divided into three etiological groups.

Variable	Group	n	Mean	SD	Median	Min.	Max.	*p*	post hoc	*p*	T.E.
RFS	LPR	43	10.65	3.04	10.00	6.00	20.00	<0.001^a^	LPR vs. Allergies	<0.001^a^	0.520
			[9.77, 11.63]		[10.00, 10.00]				LPR vs. OSA	>0.999	0.083
	Allergies	37	7.49	2.72	7.00	3.00	16.00				
			[6.65, 8.38]		[7.00, 8.00]						
	OSA	22	10.23	3.32	10.00	4.00	20.00				
			[8.95, 11.61]		[10.00, 10.00]				Allergies vs. OSA	0.002^a^	0.450

OSA, Obstructive Sleep Apnea; LPR, Laryngopharyngeal Reflux; SD, Standard Deviation; min, minimum; max, maximum; significance level *p* ≥ 0.005.

**Figure 3:**
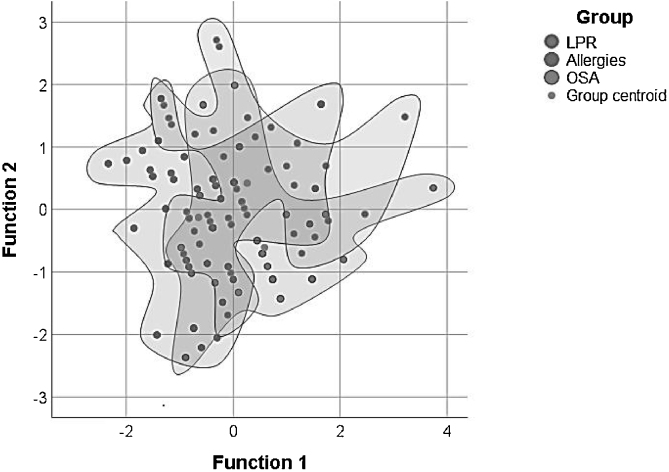
Discriminant function analysis comparing combination of positive Reflux Symptons Index(RSI) and Reflux Finding Score(RFC) of patients in the three sub-groups with chronic pharyngolaryngitis.

Using discriminant function analysis the combination of RSI and RFS scores was analyzed as a differential diagnostic strategy to try to differentiate between the three studied groups of chronic laryngopharyngitis. Interestingly it was found that the combination of both scores held a higher probability of diagnosing OSA (72.73%) and Allergies (64.86%) than diagnosing LPR (51.16%) (Table 5, Fig. 3).

**Table 5:** Discriminant function analysis results comparing combination of Reflux Symptons Index(RSI) and Reflux Finding Score(RFC) scores between groups with chronic pharyngolaryngitis.

	Group	LPR	Allergies	OSA
		n (%)	n (%)	n (%)
Original groups	LPR	22 (51.16)	13 (30.23)	8 (18.60)
	Allergies	7 (18.92)	24 (64.86)	6 (16.22)
	OSA	2 (9.09)	4 (18.18)	16 (72.73)

OSA, Obstructive Sleep Apnea; LPR, Laryngopharyngeal Reflux; significance level *p* ≥ 0.005.

## Discussion

In times of life on the fast track, fast food and fast medicine, it is essential to develop accurate, cost-effective and safe diagnostic methods for any specific disease. Chronic laryngeal and pharyngeal complaints are common in the general practitioner’s’ and otolaryngologists’ offices.^1^, ^2^ Many are the causes of such complaints.^1^, ^2^, ^3^, ^4^, ^5^, ^6^, ^7^, ^8^, ^9^, ^10^, ^11^, ^12^ Exposure to inhaled chemicals and allergens, abusive behavior, smoking, drinking, snoring and even the ever so prevalent reflux disease, are well known as irritants, and may frequently co-exist.^2^, ^5^, ^8^, ^12^ The correct diagnosis will allow effective treatment, but may be hindered by a clipped history, which will mislead the diagnosis. Most allergic adults are so well accustomed to their daily symptoms that they rarely complain unless specifically asked. Likewise, patients that snore and have sleep apnea, if sleeping alone or with a tolerant partner, may not acknowledge or even value the symptoms. The poor eating habits and unbalanced lifestyle of urban life have caused an epidemic of obesity and its consequences, such as GERD, LPR, and OSA.^8^, ^10^, ^11^ A number of studies in recent years have pointed to the possibility of an association between Laryngopharyngeal Reflux (LPR) and Obstructive Sleep Apnea (OSA).^8^, ^9^, ^10^, ^11^, ^12^, ^13^ Clinically it has been noted that LPR is more prevalent in patients with OSA than in the general population (60% vs. 20%),^8^ and that the treatment of OSA improves symptoms of LPR and vice versa.^10^, ^22^, ^23^ A positive correlation between allergic rhinitis and LPR has also been confirmed.^4^, ^5^, ^20^, ^24^ However, it is uncertain if these are only very prevalent diseases in the general adult population or if there is a direct correlation.^20^ Such a bidirectional correlation seems to have been established between food allergies and GERD.^7^

The current study elected these three common and frequently overlapping causes of chronic laryngopharyngitis to try to establish if the two most commonly used instruments for subjective diagnosis, The Reflux Symptom Index and the Reflux Finding Score, are truly capable of separating patients with GERD related laryngitis from those with other causes. Diligent investigation was carried out to create distinct groups with no apparent overlapping of etiology to their chronic laryngopharyngitis. Exclusion criteria also avoided extrinsic irritation caused by infection and chemicals (tobacco and alcohol).

One interesting finding was that patients with respiratory allergies had significant laryngopharyngeal symptoms, but less inflammatory signs on flexible nasal endoscopy, suggesting that nasal obstruction and post-nasal drip have a strong influence on symptoms such as throat clearing, sore throat, cough and dysphonia. Erin et al. also used RSI to study allergic patients with chronic laryngitis and found that the most prevalent symptom in this population was thick laryngeal mucous.^7^ No such observation as to the prevalence of a specific sub-domain was observed in the current study. On the other hand, patients that snore were more likely to present laryngopharyngeal inflammation, as observed by the higher RFS scores, but were less symptomatic. This can be explained by the mechanical trauma caused by snoring and the changes in vagal reflexes and sensibility observed in snorers. High RFS and RSI scores were observed in other studies of patients with OSA, but always looking into a positive association with LPR.^23^, ^24^, ^25^, ^26^ Patients with LPR, on the other hand, were found to have higher scores of symptoms and laryngopharyngeal inflammatory signs combined.^1^, ^2^, ^3^, ^23^, ^25^

The unexpected finding of higher probabilities of positive RSI and RFS scores in patients with OSA and Allergies was surprising. It has been well discussed in recent years how poorly specific such scores are, but they are still widely used in clinical practice for the diagnosis of LPR. It becomes evident that extra care is needed when relying solely on clinical signs to establish if the cause of the chronic laryngopharyngitis is reflux related. A careful history and physical examination, as well as other diagnostic tests to rule out respiratory allergies, OSA and other diagnosis are necessary before empirical therapeutic trials for reflux are implemented.

## Conclusions

RSI and RFS are not specific for reflux laryngitis and are more likely to induce a false diagnosis if not used diligently.

## Conflicts of interest

The authors declare no conflicts of interest.
